# Welfare technology interventions among older people living at home—A systematic review of RCT studies

**DOI:** 10.1371/journal.pdig.0000184

**Published:** 2023-01-24

**Authors:** Zada Pajalic, Diana Aguiar de Sousa, Benedicte Sørensen Strøm, Hilde Lausund, Grete Breievne, Sezer Kisa, Diana Saplacan, Marie Hamilton Larsen, Nina Jøranson

**Affiliations:** 1 VID Specialized University, Faculty of Health Sciences, Sustainable Healthcare and Welfare Technology (SHWT) Research Group, Oslo, Norway; 2 Department of Neurosciences and Mental Health, Hospital de Santa Maria, Lisbon, Portugal; 3 Lovisenberg Diaconal University College, Oslo, Norway; 4 Department of Nursing and Health Sciences, University of South-Eastern Norway, Drammen, Norway; 5 Oslo Metropolitan University, Faculty of Health Sciences, Oslo, Norway; 6 Department of Informatics, University of Oslo, Robotics and Intelligent Systems (ROBIN) Research Group, Oslo, Norway; University of Vermont, UNITED STATES

## Abstract

The main goal of health services is for the elderly to maintain their mental and physical health and live at home independently for as long as possible. Various technical welfare solutions have been introduced and tested to support an independent life. The aim of this systematic review was to examine different types of interventions and assess the effectiveness of welfare technology (WT) interventions for older people living at home. This study was prospectively registered in PROSPERO (CRD42020190316) and followed the PRISMA statement. Primary randomized control trial (RCT) studies published between 2015 and 2020 were identified through the following databases: Academic, AMED, Cochrane Reviews, EBSCOhost, EMBASE, Google Scholar, Ovid MEDLINE via PubMed, Scopus, and Web of Science. Twelve out of 687 papers met the criteria for eligibility. We used risk-of-bias assessment (RoB 2) for the included studies. Based on the RoB 2 outcomes that showed a high risk of bias (>50%) and high heterogeneity of quantitative data, we decided to narratively summarize the study characteristics, outcome measures, and implications for practice. The included studies were conducted in six countries, namely the USA, Sweden, Korea, Italy, Singapore, and the UK. One was conducted in three European countries (the Netherlands, Sweden, and Switzerland). A total of 8437 participants were sampled, and individual study sample sizes ranged from 12 to 6742. Most of the studies were two-armed RCTs, except for two that were three-armed. The duration of the welfare technology tested in the studies ranged from four weeks to six months. The employed technologies were commercial solutions, including telephones, smartphones, computers, telemonitors, and robots. The type of interventions were balance training, physical exercise and function, cognitive training, monitoring of symptoms, activation of emergency medical systems, self-care, reduction of death risk, and medical alert protection systems. The latter studies were the first of their kind and suggested that physician-led telemonitoring could reduce length of hospital stay. In summary, welfare technology seems to offer solutions to supporting elderly people at home. The results showed a wide range of uses for technologies for improving mental and physical health. All studies showed encouraging results for improving the participants’ health status.

## Introduction

### Demographic trends

The world is seeing a rapid increase in the numbers of people older than 65 [1,2]. Projections show that, by 2050 and for the first time in history, elderly people will outnumber young people [3]. According to World Population Prospects 2019, one out of six people will be over the age of 65, up from one in 11 in 2019 [2]. At the same time as this increase is occurring, the number of people working in health care is decreasing and is estimated to keep on decreasing [4,5]. The growing proportion of over-65 persons creates a challenge for providing accessible health services [4]. This challenge has resulted in the introduction of technological solutions for allowing safe and healthy living at home (e.g. information communication technology (ICT) or welfare technology (WT)) [6,7].

### Welfare technology

According to NICE 22 [8], digital health technology is classified into three levels, from A to C. Level A refers to system services and includes technologies without measurable patient outcomes and which provide services to the health care system. Level B includes communications, information, resources, public activities, patients, physicians who specialize in specific conditions, general health, and lifestyle. Level B also includes general health monitoring tools such as workout watches and symptom diaries. The technology at this level allows two-way communication between citizens, patients, and healthcare professionals. Level C is about interventions such as preventive behavior change—for example, addressing health issues such as smoking, nutrition, alcohol, sexual health, sleep, and exercise self-management. This technology enables users to manage a specific condition themselves. C-level technologies may include techniques to change behavior, or provide or guide treatment [8]. In this study, we have chosen to use the concept of welfare technology instead of digital health technology [6]. WT is an umbrella term that covers different types of technologies that enable the elderly to remain in their homes for as long as safely possible [9]. These levels of WT support the older people, their relatives, and health care professionals [10]. WT deserves our attention because it is an effective solution to secure independent living and compensates for staff shortages in health care sectors [11]. WT technologies are supposed to reduce costs and make older people more self-reliant [12].

### Older people and technology

Older people have identified independence, autonomy, and feeling safe as prerequisites for living at home, and have shown an interest in WT’s ability to allow this possibility [13]. Older people often use WT to communicate and see it as a tool that maintains relationships and reduces feelings of loneliness [14]. At the same time, these people want to continue their lives as before [4]. Therefore, telecommunication robots may enable the person to have more social contacts [15]. Similarly, companion robots are sometimes introduced into older people’s homes to provide some companionship and stimulate cognitive function [16]. However, most WT solutions have been developed for specific user groups, namely older people who have not traditionally used advanced technology [17]. Older peoples’ interactions with ICT are still limited [18]. Some do not even own a smartphone, and many feel overwhelmed by new technology and fear to use it [19,20]. Another important factor is that fewer than half of older people have computers at home. These are essential considerations when introducing these technologies into private dwellings [10,14].

At the same time, a high rate of adverse hospitalization outcomes have been documented, and it is important to evaluate strategies for improving patient self-management during the transition to home [21]. Using simply designed and user-friendly mobile technology to deliver training in the home environment can increase accessibility for many older people [22]. Furthermore, research is continuing on the use of commercially available tablets to facilitate communication in people with degenerative or chronic neurological conditions [23]. These devices may improve an individual’s memory, sense of control, communication, and overall independence [24]. Technology-assisted interventions in the home environment can also help manage chronic diseases [25]. There is emerging evidence that these relatively affordable devices have the potential to facilitate recovery as an adjunct to standard therapies [26]. This data highlights the potential benefits of recovery through the provision of interactive technology during rehabilitation [27].

Despite the potential of technology-enabled interventions, the possible negative effects of ICT cannot be ignored. For example, an overly complex user interface can lead to stress or feelings of overload, which can hinder its inclusion and acceptance [28]. If users feel that WT threatens their independence or disrupts their relationship with their primary care provider, they will not use it. Loss of privacy has been identified as a serious issue, and there are concerns about how such interventions might contribute to stigmatization and loss of autonomy [29]. Likewise, it is essential to consider how the technology is integrated into older people’s homes or other locales, e.g. hospitals, so that they are part of the decision-making process [19].

The aim of this systematic review was to examine different types of interventions and assess the effectiveness of welfare technology interventions for older people living at home.

## Methods

The systematic review of randomized control trial (RCT) interventions followed the PRISMA statement [30], guidelines of the Center for Review and Dissemination (CRD) for systematic reviews [31], and the Cochrane Handbook for Systematic Reviews of Interventions [32]. The study was prospectively [33] registered in PROSPERO [34,35] (Register number CRD42020190316).

### Data sources and search strategy

PROSPERO [34] was searched to determine whether similar studies had been completed or were still in progress. We could not find anyone registering studies with the same aim as ours.

### Eligibility criteria

The review aimed to search for peer-reviewed primary RCT studies concerning welfare technologies that have been evaluated or examined for home-dwelling persons older than 60 years. The searches were performed from May to October 2020 and additional searches were done for August 2022.

### Inclusion criteria

The following inclusion criteria were specified

- The population being studied was older (60+ years) users of technology who were living at home either alone or with other people.
- The interventions or exposures reviewed were any type of home-based technology that supports older adults in coping with everyday life.
- The technology could also be administered in various ways, including individually and through the internet.
- The control group had to have an alternative to technology for comparison.
- The types of studies considered were randomized controlled trials, controlled trials, quasi-randomized, and cluster randomized studies published as full-length peer-reviewed articles.
- Papers were published in English, Norwegian, or Swedish.
- Studies from all countries were considered.

### Exclusion criteria

The following exclusion criteria were specified:

- No target population.
- Not a primary study.
- Not published.
- Multiple publications from the same authors/projects.
- Protocol, review articles, qualitative design.
- Mixed methods, quasi-experimental studies, books.
- Studies in nursing homes and hospitals.
- Articles that did not score lower than eight on the Critical Appraisal Skills Programme (CASP).

### Information sources

A scientific librarian specializing in VID at the University of Oslo, Norway, carried out the literature search using the following electronic databases Academic, AMED, Cochrane Reviews, EBSCOhost, EMBASE, Google Scholar, Ovid MEDLINE via PubMed, Scopus, and Web of Science.

### Search strategy

Systematic searches were conducted on the studies published in English, Norwegian, and Swedish. The search strategy included a combination of the terms “intervention” AND “outcome.” The search was limited to the period between 2015 and 2020 to include the newest technology. The search strategy incorporated the following MeSH keywords in the title, abstract, and text, alone and in combination with each other: active and assisted living (AAL), ADL technologies, aging, ambient assisted living, artificial intelligence (AI), assisted living, assistive technology, autonomy, community-based, community-dwelling, e-health, e-learning, everyday technology, handheld computers, health, health informatics, healthcare robotics, home automation, independent, information and communication technology, intelligent systems, medical informatics, mobile applications, mobile devices, mobile health, mobile phones, mobile technology, older adults, own home, participation, quality of life (QoL), quantitative, RCT, self-help applications, self-help devices, self-management applications, smart home, smartphones, social, tablets, telecare, telehealth, telemedicine, telemonitoring, trials, and well-being (see example of searches in Table 1).

**Table 1:** Examples of searches in AMED.

Search for: limit 28 to yr = "2015–2020" Results: 4 Database: AMED (Allied and Complementary Medicine) <1985 to June 2020>
1 exp Aged/or aged.mp. (24056) 2 "older adults".mp. (3711) 3 Elderly.mp. (5824) 4 exp Aging/or Aging.mp. (4591) 5 assisted living.mp. (179) 6 living at home.mp. (238) 7 community living.mp. (293) 8 autonomy.mp. (1007) 9 exp Independent living/or independent living.mp. (2472) 10 social participation.mp. (329) 11 exp Communication aids/or communication aids.mp. (484) 12 exp Medical informatics/or medical informatics.mp. (771) 13 exp Telemedicine/or telemedicine.mp. (1027) 14 telehealth.mp. (212) 15 mobile phone.mp. (71) 16 smartphone.mp. (124) 17 Robotics/or robotics.mp. (585) 18 Assistive technology.mp. (545) 19 "Quality of life"/or quality of life.mp. (13799) 20 wellbeing.mp. (665) 21 QoL.mp. (1715) 22 coping.mp. (2580) 23 1 or 2 or 3 or 4 (28248) 24 5 or 6 or 7 or 8 or 9 or 10 (4216) 26 11 or 12 or 13 or 14 or 15 or 16 or 17 or 18 or 19 (3683) 28 20 or 21 or 22 or 23 (16506) 29 24 and 25 and 26 and 27 (19) 30 limit 28 to yr = "2015–2020" (4)

### Study selection and review process

**Fig 1:**
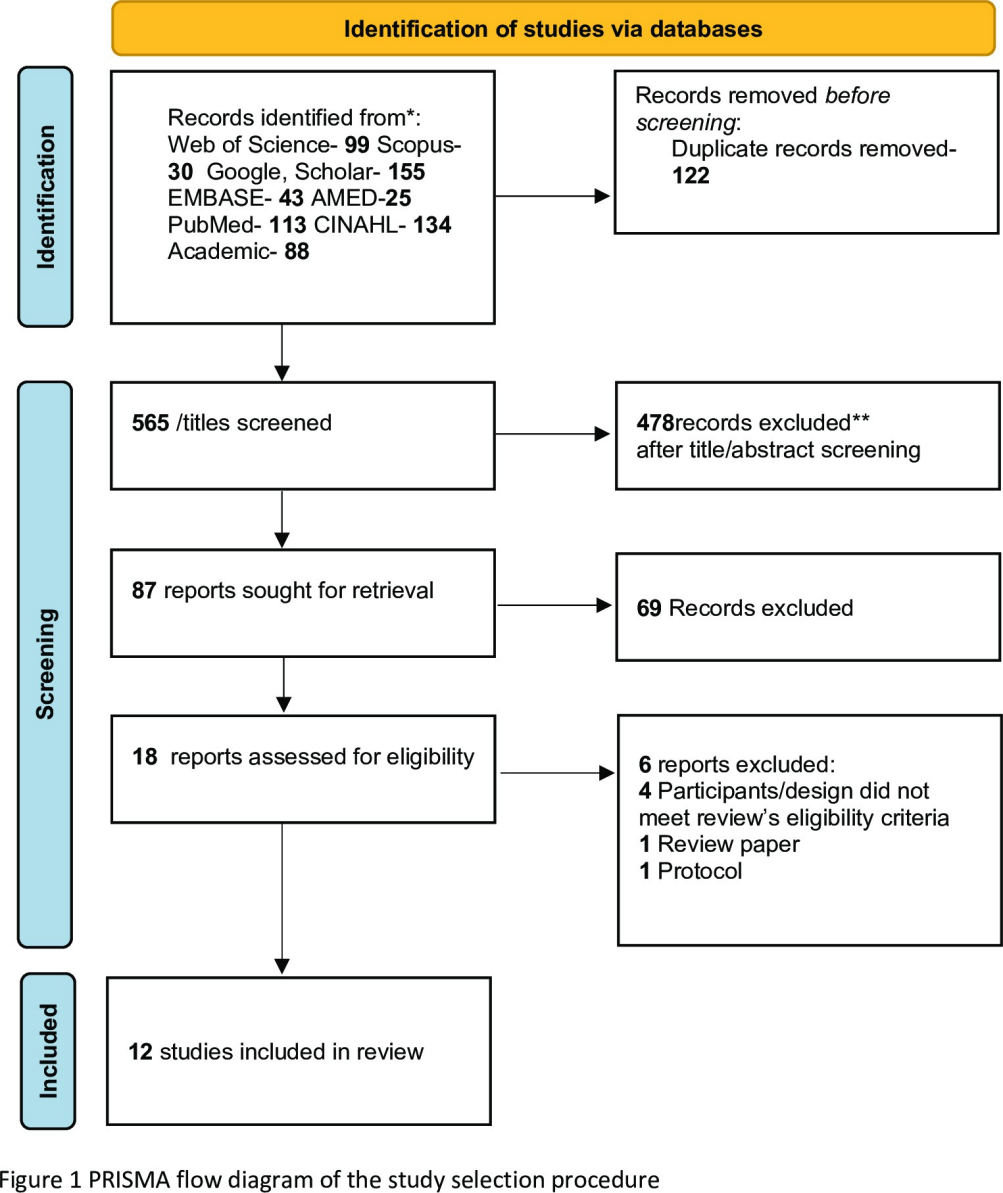
PRISMA Flow diagram here.

In total, 687 titles were identified through the literature searches. These were first imported into EndNote [36] and then into Rayyan, a web tool for comparing decisions to include or exclude studies [37]. Duplicates were removed, which brought the number down to 565 (illustrated in Fig 1 PRISMA Flow diagram). The co-authors (ZP, BSS, HL, GB, & NJ) worked together, discussed, and came to a consensus according to the following steps:

### Step 1. Screening of titles

The 565 titles were imported into Rayyan by the principal author (ZP) with blinding on, meaning that the decisions and labels of any collaborator were not visible to others, and screening was performed by all co-authors independently. The next step in the process was to invite other co-authors (BSS, GB, HL, and NJ) into Rayyan as collaborators. The authors decided to perform the first “blind on” screening independently. When the individual screening was completed, the “blind on” function was changed to “blind off,” which allowed everyone to see each other’s assessments. When it came to conflicts in independent decisions, the authors went through the titles again together. After joint discussion and consensus, 87 studies were eligible for screening of their abstracts and full text.

### Step 2. Reading abstracts and full-text articles

In this step, the authors divided themselves into three teams to read the abstracts. If the abstract was unclear or had insufficient information, the full-text article was read. This process resulted in the exclusion of 75 articles. The main reason for excluding studies was wrong population, setting, or study design. In the end, 18 studies were retained for further quality assessment (Fig 1).

### Quality assessment of papers

The CRD’s guidelines for assessing the quality [31] of the selected studies were followed in the study. The CASP RCT Standard Checklist [38] was used to appraise the selected studies and exclude those with scores lower than eight. Scores lower than nine indicated that important methodological descriptions and facts were missing. In this step, six studies were excluded for reasons including participants’ age, missing information related to randomization, incorrect design, and any change of RCT design during ongoing work. One of the studies had been corrected after publication due to disagreement between the researcher and the owners of the technology, and it was not possible to determine how this conflict had affected the study’s outcome from the corrigendum. This study was therefore excluded. Finally, 12 articles were included in the review [39–50] The CASP assessment of included studies is illustrated in Table 2.

**Table 2:** CASP assessment of included studies [38].

CASP	Q1	Q2	Q3	Q4	Q5	Q6	Q7	Q8	Q9	Q10	Q11	Total
Bao et al.	1	1	1	0	1	1	1	X	1	1	1	9
Corbett et al.	1	1	1	1	1	1	1	1	1	1	1	11
Hong et al.	1	1	1	X	1	1	1	1	X	1	1	9
Kim et al.	1	1	1	X	1	1	1	1	X	1	1	9
Ong et al.	1	1	1	0	1	1	1	1	1	X	X	8
Mavandadi et al.	1	1	1	0	1	1	1	1	1	1	1	10
Matz-Costa et al.	1	1	1	X	1	1	1	1	1	X	1	9
Melin et al.	1	1	1	1	1	1	1	1	1	X	X	9
Morgenstern et al.	1	1	1	0	1	1	1	1	1	1	1	10
Pedone et al.	1	1	1	0	1	1	1	1	1	1	X	9
Radder et al.	1	1	1	X	1	1	1	1	1	1	X	9
Simon et al.	1	1	1	0	1	1	1	1	1	1	1	10

**CASP checklist:** Q1. Did the study address a clearly focused research question? Q2. Was the assignment of participants to interventions randomized? Q3. Were all participants who entered the study accounted for at its conclusion? Q4. Were the participants “blind” to the intervention they were given? Were the investigators “blind” to the intervention they were giving to participants? Were the people assessing/analyzing outcome/s “blinded?” Q5. Were the study groups similar at the start of the RCT? Q6. Apart from the experimental intervention, did each study group receive the same level of care (that is, were they treated equally)? Q7. Were the effects of intervention reported comprehensively? Q8. Was the precision of the estimate of the intervention or treatment effect reported? Q9. Do the benefits of the experimental intervention outweigh the harms and costs? Q10. Can the results be applied to your local population/in your context? Q11. Would the experimental intervention provide greater value to the people in your care than any of the existing interventions?

Answers: 1 = Yes; 0 = No; X = Unclear [38].

**Fig 2:**
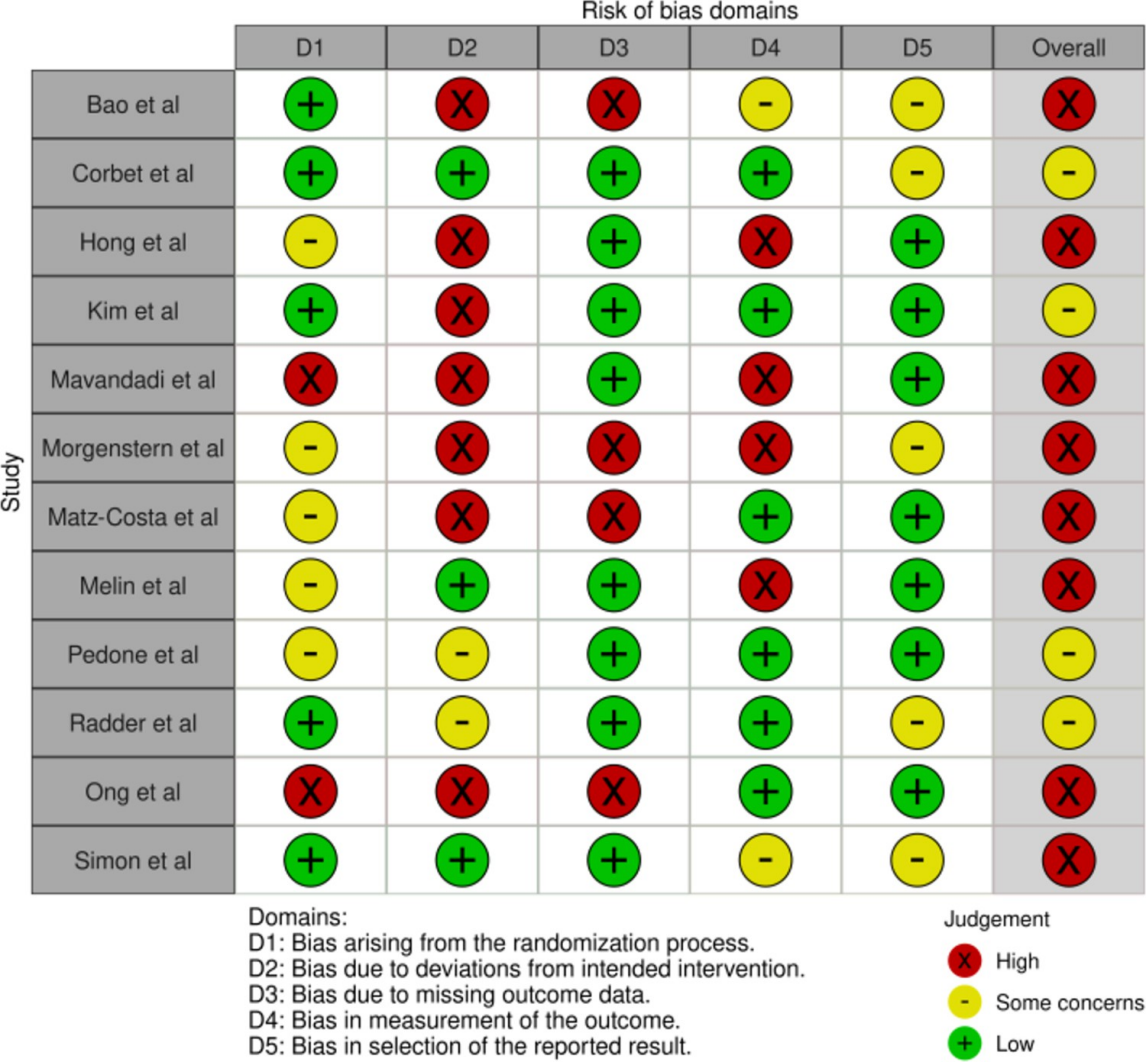
RoB 2 Traffic-light plot of included studies here.

**Fig 3:**
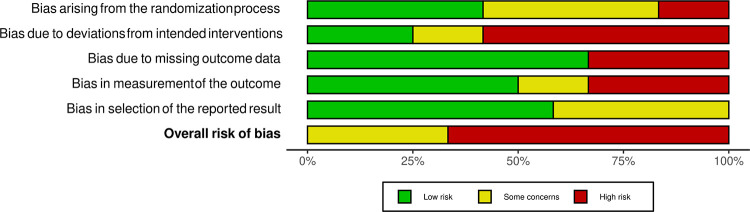
RoB 2 Summary plot of included studies here.

There was significant variation among the included studies in terms of design, type of intervention, selected participants, and intervention outcomes. Before the next step, the 12 selected articles underwent a risk-of-bias assessment for randomized trials (RoB 2) [51,52], which was performed by three co-authors (SK, MHL, & ZP). The outcomes of the RoB 2 screening showed that meta-analysis was not possible due to high heterogeneity in the included studies. Results of the RoB 2 analysis are illustrated in Figs 2 and 3. Based on the RoB 2 outcomes and high heterogeneity of the quantitative data, a narrative summary [53] was chosen to provide the study characteristics, outcome measures, and implications for practice. The included studies are presented in Table 3, which is an overview.

**Table 3:** Overview of included studies.

Author, year, country	Design of study	No. of arms or groups (including control groups)	Type of technology and Tier classification	Duration of intervention	Purpose of technology/ type of intervention	No. of participants in EG, IG, CG, MA, and CM	Data collection	Analysis	Primary outcome/s	Implication for practice
Bao et al., 2018, USA	RCT	2 arms	Smart phone balance trainer Tier 3	8 weeks	Balance training	EG n = 6 CG n = 6	ABC; SOT; Mini-BESTest; 5xSST; FSST, FRT, 10mWT, TUG, TUG-COG	Mean, SD, two tailed student’s t-test, linear mixed model, Bonferroni corrections for paired t-tests	Pre-mid-post-training clinical balance testing (CBT)	Study supports smartphone as a balance rehabilitation and potential telerehabilitation tool for use by healthy community dwelling older adults
Corbett et al., 2015, UK	RCT	3 arms	On-line training package Tier 2	6 months	Cognitive training	EG ReaCT n = 2557 CGT n = 2432 CG n = 1753	IADL scale, Baddeley Grammatical Reasoning test, SWM “digit span” task, paired associates learning test	Mixed-effects regression mode, post hoc analyses	Self-reported IADL	Online CT confers significant benefit to cognition and function in older adults and has potential as a public health intervention
Hong et al., 2017, Republic of Korea	RCT	2 arms	PC and video conferencing by Skype Tier 2	12 weeks	Tele-exercise	EG n = 11 CG n = 12	Height, weight, body fat %, upper and lower limb muscle mass, and ALST	Independent t-test, Kolmogorov-Smirnov one-sample test, two-way repeated ANOVA	Body composition and functional fitness	Tele-exercise seems to be effective intervention method for preventing or improving sarcopenia
Kim et al., 2015, Republic of Korea	RCT	2 arms	Robot-assisted training Tier 3	12 weeks	Cognitive training	EG n = 48 CG n = 37	ADAs; the seven subtests from the CANTAB; DMS; PRM; PAL; SWM; SOC; RTI; RVIP; MRI (3D T1 TFE, FLAIR, T1 REF, T2, FFE, and DTI)	Student t-test, Chi-square, general linear model (GLM, paired t-test,	Changes in cortical thickness as assessed by surface based morphometry (SBM),	Robot-assisted cognitive training can help improve cortical thinning in the elderly
Mavandadi et al., 2015, USA	RCT	2 arms	Telephone Tier 2	6 months	Symptom monitoring	MA n = 377 CM n = 401	BOMCT; Mini-International Neuropsychiatric Interview; PHQ-9; 5-item Paykel Scale for suicide ideation, SF-12; GAD-7; MSC	χ2, 2-sided test, g intent-to-treat, mixed-effects linear regression models	12-Item Short-Form Health Survey MCS score	Telephone delivered symptom monitoring is feasible and can be delivered across a large geographical region in a scalable manner using informatics support and available individual data for case finding
Morgenstern et al., 2015, USA	Pilot RCT	2 arms	Medical monitoring assistance device Tier 1	90 days	Activation of emergency medical systems	EG n = 112 CG n = 122	Demographics; contact information; social isolation/connectedness scale; anxiety; depression; stroke knowledge questions; HRQOL (HADS)	ANOVA, means, SD, t-test, Chi-squared tests, simple linear regression	Number of healthy days	Technology may be a solution to activating emergency medical systems for time-limited acute medical conditions like stroke
Matz-Costa et al., 2018, USA	RCT	2 arms	Pedometers, tablet, and peer mentoring via phone Tier 3	8 weeks	To embed physical activity, cognitive activity, and social interaction	EG n = 12 CG n = 13	Feasibility measures; PA; no. steps per day; CA; SI; PM	T-test, Chi-square tests, multilevel mixed-effects linear regression, unstandardized coefficients associated with treatment-by-time interactions using methods by Feingold	Physical activity (PA) (no. steps per day)	T intervention shows promise in increasing steps per day compared to the CG over both a 4-week and an 8-week period
Melin et al., 2018, Sweden	RCT	2 arms	Tablet computer Tier 3	6 months	Effects on self-care, Quality of Life, and Knowledge	IG n = 66 CG n = 70	EHFScBS-9; KCCQ; Swedish version of the HSDHFKS	χ2, test, and a t-test, generalized Poisson log-linear regression, Mann-Whitney U test	Self-care behavior	Software system significantly improves self-care behavior and health related quality of life, increases knowledge of heart failure, and reduces users’ hospital days
Pedone et al., 2015, Italy	RCT	2 arms	Tele-monitoring Tier 1	6 months	Comparing multiparametric tele-monitoring plus telephonic support with standard care	IG n = 50 CG n = 46	ADL; IADL; NYHA; CIRS; Serum concentration of NT-proBNP, inhibitors (ACE-Is) and angiotensin receptor blockers (ARB)	Descriptive statistics and t-tests or Chi-square tests, product-limit method complemented with the log-rank test, proportional hazards model,	Hospital admissions for any reason or death 180 days from enrollment	Physician-led tele-monitoring of elderly adults with HF is feasible and reduces the risk of death and hospitalization
Radder et al., 2019, Netherlands, Sweden, Switzerland	RCT	3 arms	ironHand Tier 3	4 weeks	Improving hand function	EG1 n = 25 EG2 n = 22 CG n = 30	Maximal handgrip strength; Maximal pinch strength; JTHFT; BBT; SUS; use time	Descriptive statistics, using mean±SEM, One-Way ANOVA, Chi-square test or the Fisher exact test, mixed-model analysis, Wilcoxon signed rank test, Mann-Whitney U test	Handgrip strength	Wearable robotics as provided through the ironHand system may improve functional performance in persons with hand problems associated with aging in general
Ong et al., 2017, Singapore	RCT	2 arms	Medical Alert Protection Systems (MAPS) and eAlert Tier 2	6 months	Revisit reduction to ED, reduction in the number of hospitalizations, and reduction in total length of stay of admissions	EG n = 72 CG n = 90	Reduction in ED visit rate compared to baseline; reduction in number of hospitalizations compared to baseline; total length of stay for admitted patients, self-reported confidence; EQ-5D	Percentages, median, Interquartile Range (IQR) and range, chi-square test, and Mann-Whitney test	Number of ED visits, number of hospital admissions, and length of stay at hospital	Use of the MAPS resulted in a reduced total length of stays for patients with one or more admissions
Simon et al., 2018, USA, Sweden	RCT	2 arms	Computerized program for cognitive training Tier 2	5 weeks	To evaluate the efficacy of CCT focused on WM compared to an active control condition	EG n = 37 CG n = 40	SWM; TMT-A; TMT-B; WAIS-IV; COWAT; labeled Phonemic Fluency, Semantic Fluency	Pearson’s chi-square test, t-test, Linear Mixed Models, Restricted Maximum Likelihood Method, Benjamini-Hochberg procedure, ANOVA	Performance changes on Cogmed tasks, training effects	Healthy older adults from different cultural backgrounds can benefit from a home-based intensive five-week computerized working memory training

**Abbreviations:** ABC = Activity Specific Balance Confidence, ADL = Activities of Daily Living, ADAS = Alzheimer’s Disease Assessment Scale, ALST = Appendicular Lean Soft Tissue Test, BBT = Box and Blocks Test, BOMCT = Blessed Orientation-Memory Concentration Test, CA = Cognitive Activity, CANTAB = Cambridge Neuropsychological Test, CBT = Clinical Balance Testing, CG = Control Group, CIRS = Cumulative Illness Rating Scale, CM = Case Management, COWAT = Controlled Oral Word Association Test, DMS = Delayed Matching to Sample, ED = Emergency Department, EG = Experimental Group, EHFScBS-9 = 9-item European Heart Failure Self-care Behavior Scale, EQ-6D = Health Related Quality of Life, 5xSST = Five Times Sit to Stand Test, FRT = Functional Reach Test, FSST = Four Square Step Test, GAD-7 = Generalized Anxiety Symptom Severity, HADS = Hospital Anxiety and Depression Scale, HF = Heart Failure, HRQOL = Health Related Quality of Life, HSDHFKS = Health Survey Dutch Heart Failure Knowledge Scale, IADL = Instrumental Activities of Daily Living, IG = Intervention Group, JTHFT = Jebsen-Taylor Hand Function Test, KCCQ = Kansas City Cardiomyopathy Questionnaire, MA = Monitoring Alone, Mini-BESTest = Mini Balance Evaluation Systems Test, MSC = Mental Component Subscale, NYHA = New York Heart Association, PA = Physical Activity, PACE = Pharmaceutical Assistance Contract for the Elderly, PACENET = Pharmaceutical Assistance Contract for the Elderly Needs Enhancement Tier, PAL = Paired Associates Learning, PM = Personal Meaning, PRM = Pattern Recognition Memory, RTI = Reaction Time, RVIP = Rapid Visual Information Processing, SEM = Standard Error of the Mean, SF-12 = Short-Form Health Survey, SI = Social Interaction, SOC = Stockings of Cambridge, SOT = Sensory Organization Test, SUS = System Usability Scale, SUSTAIN = Supporting Seniors Receiving Treatment and Intervention, SWM = Spatial Working Memory, 10mWT = Ten-meter Walk Test, TMT-A = Trail Making Test Part A, TMT-B = Trail Making Test Part B, TUG = Timed UP and Go, TUG-COG = Time Up and Go with Cognitive Task, WAIS-IV 4^th^ ed = Weschler Adult Intelligence Scale–Fourth Edition, WM = Working Memory.

## Results

There appeared to be great variation among the selected studies in terms of design, type of intervention, selected participants, and intervention outcome. Based on the RoB 2 outcomes, the overall risk of bias was high (65%) as was the heterogeneity of the quantitative data. Due to high risk of bias it was therefore decided to narratively summarize the study characteristics, outcome measures, and implications for practice.

All 12 studies were published during the last seven years 2015–2019 and were conducted in six countries, namely the USA [39,43,44,46], Sweden [45,50], Korea [41,42], Italy [48], Singapore [47], and the UK [40]. One study was conducted in three European countries (the Netherlands, Sweden, and Switzerland) [49]. In total, 8437 participants were sampled, and individual study sizes ranged from 12 [39] to 6742 [40]. Most studies were two-armed RCTs, except for two that were three-armed [40,49].

The duration of the interventions in the studies varied. The shortest interventions were four weeks [49] and five weeks [50]. Other interventions lasted eight weeks [39,43], twelve weeks [41,42], and ninety days [46]; the longest intervention of six months was applied in five studies [40,44,45,47,48].

The technologies applied were mostly commercial solutions in the form of telephones [44], smartphones [39], computers [41,45], telemonitoring [46,48] and robots [42,49].

The types of interventions varied and included balance training, physical exercise and function [39,41,43,49,50] cognitive training [40,42], monitoring of symptoms [44], activation of emergency medical systems [46], self-care [45], reduction of risk of death, and total length of stay at the hospital [47,48].

Categories of outcomes related to the interventions varied across the studies. Physical activity and training were assessed through balance testing [39], body composition testing [41] daily steps [43], or handgrip [49]. Cognitive training was assessed by measuring cortical thickness [42] or task performance [50]. Health monitoring was assessed by health survey forms [46], emergency system activation by the number of healthy days [44], and monitoring of self-care [40]. Reduction of hospital readmission was assessed by the number of days to readmission [47,48].

Interventions in the form of clinical balance testing with a smartphone, which affects self-care behavior and the health-related quality of life test, online cognitive training, and remote training, appeared to be effective intervention methods [39–41]. The ironHand system may help people with hand problems [49].

Robot-assisted cognitive training may improve cortical thickness in the elderly. Symptoms can be monitored via telephone, and emergency systems exist for time-limited acute medical conditions. Physician-led telemonitoring of older adults with heart failure is shown to be feasible and reduces the risk of hospitalization and death [47,50].

The use of Medical Alert Protection Systems (MAPS) reduced the total length of stay for patients with one or more hospital admissions. Other studies showed that healthy older adults of diverse cultural backgrounds can benefit from an intensive home-based five-week computerized working memory training [45,50].

The studies showed notable effects of WT interventions on physical and psychological health status. However, due to the high risk of bias, it was not possible to draw any significant conclusions.

## Discussion

Our study indicates that WT can be used to improve people’s health. Remote digital support has been shown to impact older adults’ health and function significantly. Welfare technology can be used as a tool for health-related interventions to improve the health of the individual or group at the primary, secondary, and tertiary levels. Interventions using WT can cover a large geographical area and be available to many older adults simultaneously over a short or long time and around the clock. Our results are supported by the study of Holthe et al. [54] who are optimistic about the effectiveness of supportive technology for community-dwelling older adults. Although WT is promising, there are still challenges with the user-friendliness of different solutions that end-users must consider [54]. This was evident in the Bhattarai et al. study, which showed the importance of including older people with arthritis in all design and development stages for WT solutions [55].

Participatory design (PD), a Scandinavian-anchored methodology, should be considered when developing technologies to be used by, for, and with end users (elderly persons, people who are less than technically savvy, people unfamiliar with new technology, or even “novices”). Several studies have shown the benefits of end users’ involvement in the co-design of technology [56,57]. Similarly, the user’s physical limitations [19,20,58,59] should be considered when designing technology for older people. The design of WT can enhance or diminish older users’ abilities. A good design will improve their abilities regardless of their health situation, including cognitive limitations or reduced physical ability, such as hand tremors [20].

This study showed that interventions focusing on outcomes such as self-care behavior and health-related quality of life appear to be effective, which is demonstrated by the system focusing on functional performance [39–41]. Our results confirmed that such interventions also showed a positive impact among patients with heart failure who received help to better control their sodium intake [60]. Gallucci et al.’s study points out that research examining ICT use among older people living at home is still in the testing phase [61]. This study confirms the results of our study, which indicates that intervention studies should clearly define older people’s needs and the expected impact of ICT on health to critically assess the implementation of different ICT solutions in the context of home care [61]. Several studies have investigated the effects of various interventions on home-dwelling older people. An American RCT found some benefit after improving telemonitoring of heart failure among older adults after six months [62]. Another study found that telecare was no better than traditional care for patients with chronic obstructive pulmonary disease, and found no significant effects on health-related quality of life after 12 [63]. The diversity of findings in these RCTs could explain some of the challenges in conducting high-quality studies when investigating interventions with telecare to such a heterogeneous population as elderly people. There is still a lack of rigorous evaluation and a need to explore users’ experiences with these technologies to design more innovative and tailored RCTs [62]. In our study, it was shown that the use of WT for cognitive training and symptom monitoring had promising results in terms of reducing risk of death and hospitalization. Our results are confirmed by Meiland et al. [64] who highlighted the use of WT in the health and care sectors, specifically focusing on older people living in their own homes [64].

Different measures need to be prioritized, such as development, usability, efficiency, cost-effectiveness, deployment, and ethics for assistive technology and health technicians. It is important to stop replicating technology that is unhelpful or inefficient. Further, it is essential to focus on how technologies succeed in meeting the needs of older people with various diseases. Collaboration between practitioners, decision-makers, health insurers, and caregivers working together with technology companies and researchers is vital for developing strategies for implementing aids in different care environments. These strategies can help future generations use available and affordable technology and ultimately encourage the acceptance of WT among older people [61,64]. Peek et al. [65] looked at the implementation of technology for older people and found that different kinds of technology were appropriate to enable a more independent life, depending on the purpose of the technology and the conditions in which it was implemented [65]. Furthermore, the importance of prioritizing users’ needs and acceptance are emphasized. Of particular importance is tailoring technology to the specific needs of each user. Universal Design and its principles [66], along with accessibility and usability aspects, modularity, adaptability, and customization of technology may become more important in the near future. Further implementation of WT is a complex process that must involve attitude changes at different organizational levels in the health and care sectors, policy changes, cross-organizational cooperation, interdisciplinary education, and continuous development and refinement of WT and careful evaluation of its impact on users’ health [65].

In our study, it was shown that healthy older adults of diverse cultural backgrounds can benefit from a home-based, intensive, five-week computerized working memory training program [45,50]. Acceptance of WT among older people depends on their previous experience of using technology, how well they understand its functions, and whether they feel confident as users [67]. The challenge of WT is that it is also a generational issue, especially regarding ICT literacy: older people can have a hard time learning procedures or absorbing instructions, especially if they are no written in their native language [68]. This finding is confirmed in other studies. For instance, elderly persons prefer to interact with technology that uses both their native language and non-technical wording [19,20,58,59]. Similarly, older adults have shown to prefer interacting with technology through speech rather than visually. An ethical challenge of testing WT among frail older people is that the WT used in home tends to be similar to that found in hospitals or care homes, making the home environment an extension of the institution. Such processing tends to reduce older people to passive subjects of data monitoring. Legal and ethical concerns include loss of privacy and uncertainty about where data is stored and who can access it [69]. Other studies discuss ethical challenges such as how older people may lose some human contact when technology is their sole source of social interaction. They may also feel objectified and lacking control, along with feelings of betrayal and infantilization; they may also worry about who is responsible for the technology and their care—whether their “care” is at the mercy of the technology, or whether there is still a human in the loop [28].

This study shows ways in which WT is used by a group whose conditions, needs, and abilities are changing. WT should be designed with some degree of flexibility to meet different degrees of disability. The most important thing is that WT be user-friendly and adapted for use in the home. Technology is always changing, which is a challenge for the older people who use it. More studies addressing this challenge are needed.

### Strengths and limitations

The method we implemented is in line with PROSPERO’s criteria and the Prisma guidelines to ensure that they fell within the scope of the study and that the necessary data was provided. Published articles were identified in the databases with the help of the research librarian and co-authors, who are experts in their field. Searches were limited to the Nordic languages ​and English. Several studies had small sample sizes. This is one weakness of the study, which makes generalization of the results impossible. A review of articles was conducted by five co-authors independently and then in dialogue and consensus. One of the study’s strengths is the quality assessment using the CASP checklist and then the RoB 2 for bias control, which was performed by three co-authors with expert knowledge.

## Conclusion

This study shows a wide variety of different WT solutions used to influence the health status of older people who live at home. Regardless of the length of the intervention, all studies showed WT’s potential for improving the health of the elderly, empowering them, and reducing the workload on health care providers. In addition, the studies identified concerns regarding the risk of bias, indicating the need for more robust studies whose results can be implemented in similar contexts.
